# Ecological Drivers of Breeding Peak in Hamadryas Baboons (*Papio hamadryas*) Across Two Contrasting Habitats in Saudi Arabia

**DOI:** 10.3390/ani16172724

**Published:** 2026-09-02

**Authors:** Ahmed Boug, Zaffar Rais Mir, Toshitaka Iwamoto, Mengjing Wei

**Affiliations:** 1 National Center for Wildlife, Riyadh 12411, Saudi Arabia; zaffarmir@ncw.gov.sa (Z.R.M.); w.mengjing@ncw.gov.sa (M.W.); 2 Faculty of Education and Culture, University of Miyazaki, Miyazaki 889-2192, Japan; iwamot.t@gmail.com

**Keywords:** hamadryas baboon, reproductive peak, estrous, ecological factors, Saudi Arabia

## Abstract

Reproduction could be the most important control point for the spread of hamadryas baboons (*Papio hamadryas*). Understanding the drivers of reproduction is necessary to determine which interventions may work best, in which locations, and at what time of year. We analyzed monthly variation in the proportion of estrous females and black infants in two troops inhabiting two different sites (Dam and Al Hada) in Saudi Arabia over the course of one year (one week per month; ~720 h per site-year; non-contemporaneous). The effects of season, rainfall, natural food diversity, and anthropogenic foods were studied using generalized linear models with additive nonlinear terms. At the hotter and drier Dam site, seasonality explained most variation, with a higher proportion of estrous females observed during the hot season. At the cooler and wetter Al Hada site, infant proportions and estrous females responded nonlinearly to rainfall, natural food diversity, and human food subsidies, with less seasonality. Patterns in the proportion of infants broadly aligned with the estrous data and provided insight into the temporal distribution of births across the two troops. The two-troop contrast in this study suggests reproductive flexibility. Specifically, there may be a dual control system with an intrinsic seasonal control modulated by a flexible ecological response. Further work is needed to understand the scales and conditioning of environmental drivers of baboon reproduction to inform wildlife management.

## Simple Summary

Hamadryas baboons increasingly come into conflict with people in Saudi Arabia, yet little is known about when they breed or how local conditions influence breeding. We compared one year of observations from two troops living in contrasting environments in western Saudi Arabia. Each month, we recorded the percentage of females showing visible signs that they were ready to mate and the percentage of young infants with black coats, then examined how these patterns related to season, rainfall, natural food variety, and food provided by people. At the hotter and drier Dam site, more females were ready to mate during the hot season, while black-coated infants were most common in the cooler season. At the cooler and wetter Al Hada site, the reproductive patterns were less strongly seasonal and appeared to vary with rainfall, natural food variety, and the use of human-derived food. These findings show that breeding in hamadryas baboons can shift with the local environmental conditions rather than following one fixed yearly pattern. This knowledge may help wildlife managers better time waste control, public awareness, and other measures intended to reduce human–baboon conflict.

## 1. Introduction

Reproductive cycles across species are often time-structured in many non-human primates, with populations exhibiting marked annual patterns in conception and birth that reflect environmental and social constraints [1,2,3,4]. The timing of conception and birth reflects interactions among rainfall, food availability, temperature, photoperiod, social conditions, and intrinsic physiological rhythms [2,5,6]. Though seasonality in baboon reproduction is well documented [4,7], the proximate mechanisms influencing birth periodicity are often unclear [8] or difficult to clarify [9]. Despite this uncertainty, food availability has started to emerge as an important driver for the timing of births [3,10]. Greater food availability during the ovarian cycle appears to increase the probability of conception, as well as facilitate the storage of body reserves for lactation and gestation [8]. The birth season appears to be timed to when the females can obtain more surplus energy and protein [11,12]. Experimental work in Japanese macaques (*Macaca fucata*) further shows that photoperiod alone may be insufficient to initiate ovarian activity; temperature, social cues, and seasonal changes in hypothalamic-pituitary sensitivity can interact with endogenous rhythms [13,14]. Long-term work on yellow baboons (*Papio cynocephalus*) documented broad variation in reproductive and infant-survival parameters rather than obligate seasonal breeding [15], while olive baboons (*Papio anubis*) at Gilgil showed annual birth patterns that varied over a decade [8]. Other populations provide further within-genus contrasts: high-altitude Drakensberg chacma baboons showed a clearer birth peak, whereas most conceptions among chacma baboons (*Papio ursinus*) in the seasonally flooded Okavango Delta occurred during the period of highest rainfall [10,16]. Together with the comparatively diffuse reproduction reported at Gilgil, these patterns show that reproductive timing varies with rainfall regime, altitude-associated climate, and habitat seasonality even within *Papio* [3,8]. Thus, reproductive timing is best interpreted as the outcome of multiple proximate cues rather than as a fixed response to one environmental variable.

Despite widespread variability in reproductive patterns across Cercopithecinae and *Papio*, some reports on hamadryas baboon (*Papio hamadryas*) describe no distinct birth peak in the species (*Papio hamadryas*) [7,17]. By contrast, a study in Ethiopia reported bimodal birth peaks in the species, with elevations in early summer (May–June) and early winter (Nov–Dec) [18]. No follow-up work has been able to validate this pattern, nor is the bimodal pattern reported in other species to the best of our knowledge [18]. Without data over at least a multiyear period, it is difficult to know for certain whether there is a temporal pattern to births among wild hamadryas. Notably, a 30-year study of a food-provisioned colony demonstrated moderate seasonality in ovarian activity despite stable food supply, with conceptions peaking in spring/early summer and births concentrated in winter—consistent with a capital-breeding strategy in which stored reserves buffer seasonal energetic shortfalls [19]. This could suggest that food availability is relevant but not fully dominant in shaping reproductive timing for hamadryas baboon. The understanding of reproductive timing in hamadryas baboon remains cryptic, and more is understood about the basic reproductive physiology than the ecology or temporal structuring.

Female primates exhibit short, cyclic estrous periods driven by ovarian hormone fluctuations, and these periods are accompanied by behavioral signals that reliably indicate fertility to males [20,21,22]. Among anthropoid primates, behavioral estrus is often decoupled from ovulation in that mating activity also occurs during non-fertile phases of a female’s cycle [20]. Many primate taxa, including baboons, show physiological signs of estrus, such as sexual swellings and face color changes that correspond with behavioral estrus. In baboons (*P. h. anubis* and *P. h. cynocephalus*), sexual swellings have been shown to correspond closely with levels of ovarian hormones and to occur during the period just before, during, and just after ovulation [23]. Life-history parameters further contextualize reproductive timing in hamadryas. Sigg et al. (1982) report that female hamadryas baboons at Erer Gota give birth to their first infant at an age of about six (mean 6.1, range 5.5–7, n = 8), suggesting that first conception in wild hamadryas occurs at an average age of about five and a half years, about a year after menarche. Female hamadryas baboons characteristically have an estrous cycle of 31 to 35 days in length [17]. At Filoha, the reported mean interbirth interval was 22 months (n = 12) [7]. These relatively long reproductive intervals emphasize the need for multiyear observations when stable population-level seasonality is the primary question.

In more than 30% of primate species, one of the most noticeable and widespread morphological developmental traits is the transition from natal fur and skin color to adult coloration [24,25,26]. In hamadryas baboons, infants are born with black fur, which gradually changes to a yellowish-brown color after approximately six months. This pattern has also been observed in other baboon species, such as yellow baboons [27,28] and chacma baboons [29]. The variation in fur coloration between individuals can be used as a distinguishing characteristic for identifying and differentiating juveniles within their social groups.

Despite important advances in understanding primate reproductive timing, most research on hamadryas baboons has been conducted in the Horn of Africa [7,17,18], and quantitative data on estrous and birth timing from the Arabian Peninsula are extremely limited. In particular, there is a lack of multiyear or cross-cohort datasets from Saudi Arabia that combine systematic behavioral sampling of estrous females with detailed measures of natural and anthropogenic food availability, rainfall, and plant community structure.

Human–baboon conflict is a key wildlife management concern in Saudi Arabia. Roadside feeding, poorly secured waste, agricultural foods, and other subsidies can attract baboons into close contact with people, contributing to crop and property damage, traffic and public-safety concerns, and risks to baboon welfare [30,31,32,33]. In a survey of 1356 residents across six affected areas, 63% reported daily baboon incursions into farms or homes, 60% reported damage to crops or property, and 67% expressed negative attitudes towards baboons; reported losses ranged from US$250 to more than US$1300 per household per year [30]. Similar processes elsewhere show that food-related raiding can become established through repeated access to high-value anthropogenic resources [33,34]. Understanding reproductive rates and their drivers is central to prioritizing and selecting the most effective interventions (e.g., fertility control, reducing access to anthropogenic food, habitat-based management, or targeted management of conflict-prone groups). Moreover, understanding how food interacts with breeding may be important for spatially explicit management and for predicting nonlinear interactions between food resources and reproduction. The extent to which there is one annual reproductive peak or two also informs how much of the year and when in the year interventions should target reproduction, and when such efforts might be misguided.

Here, we evaluate the reproductive histories of two hamadryas baboon troops in western Saudi Arabia. One is located on the wetter windward escarpment of the Sarawat Mountains (Al Hada), and the other is located in the drier eastern rain shadow (Dam site). Human activity also differs between sites, with substantially higher tourist and recreational footfall at Al Hada, while the Dam site is comparatively less disturbed but ecologically harsher. Predator communities may also have differed between sites, with scavengers and large carnivores such as striped hyenas appearing more abundant around Al Hada, likely reflecting greater prey availability and landscape connectivity. Our aim is to provide the first detailed comparative assessment of within-year variation in reproductive activity in hamadryas baboon troops in Saudi Arabia and to clarify the ecological drivers and correlates of the timing of breeding in this species. Although collected a long time before, these data provide a rare year-round baseline of hamadryas baboon reproductive timing prior to recent landscape and management changes in western Saudi Arabia. Accordingly, our aim was to compare ecological correlates of within-year reproductive timing, rather than to test reproductive synchrony between the two populations or to attribute all observed site differences to habitat alone. Because the historical datasets were collected approximately 10 years apart, potential temporal changes in environmental conditions, population structure, and management context cannot be fully excluded, and this temporal separation is therefore explicitly acknowledged as a limitation. Nevertheless, because the data captures intrinsic behavioral and reproductive patterns across the annual cycle, their relevance remains robust despite subsequent population changes. Understanding the temporal aspects of reproduction in baboons is expected to allow managers to predict periods of heightened energetic demand and risk-taking behavior, enabling timely, targeted, and cost-effective human–baboon conflict mitigation.

## 2. Methods

### 2.1. Study Area

We conducted this study at two sites in the central-western part of Saudi Arabia, both of which support significant populations of hamadryas baboons: (1) Al Hada escarpment (21.37127° N, 40.24964° E) and (2) the Dam site (21.21844° N, 40.45381° E). The data collection periods were 1990 for Al Hada and 2000 for the Dam site.

**Figure 1:**
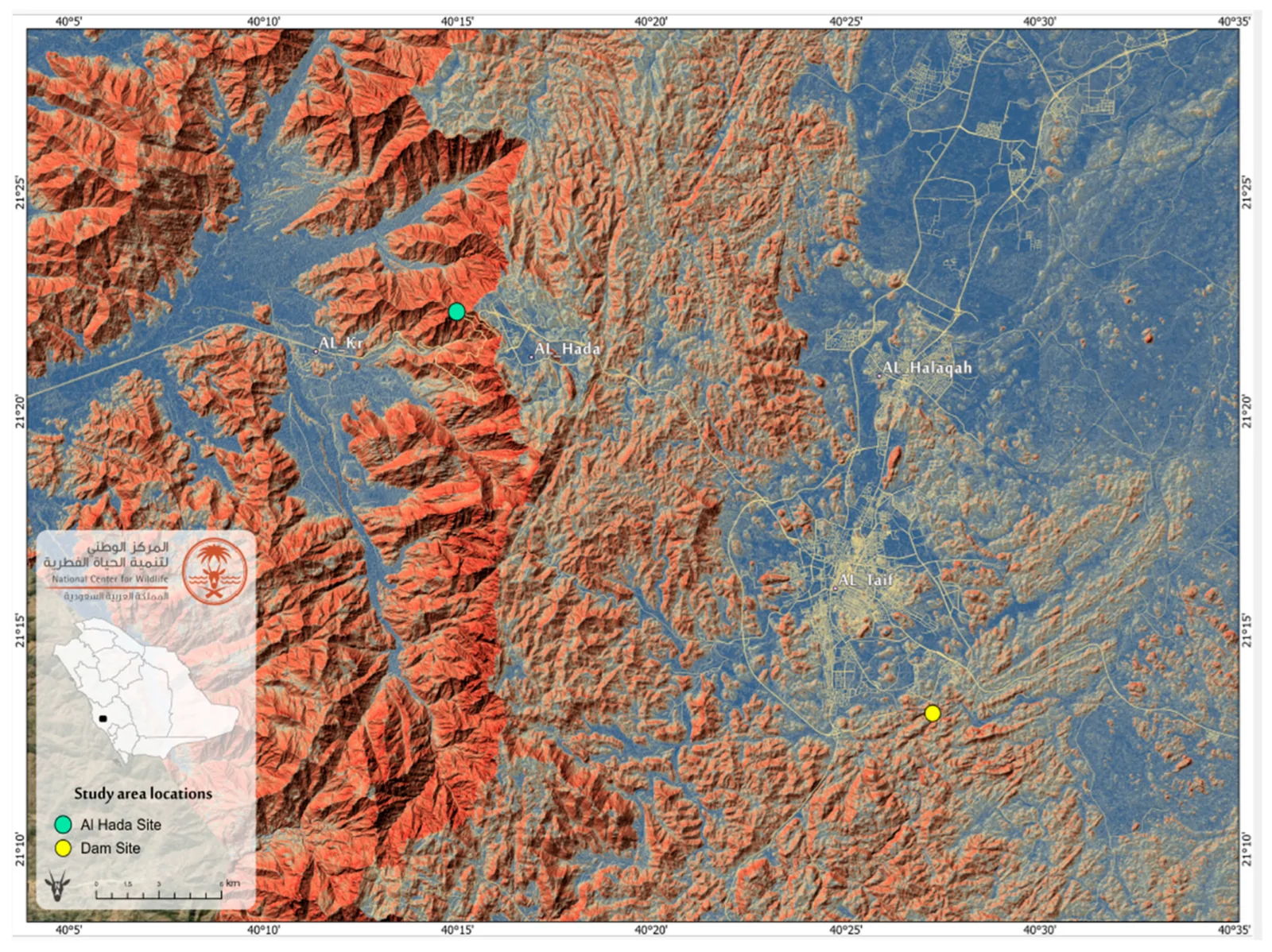
Location of the two hamadryas baboon study sites in western Saudi Arabia. The Al Hada site (green) lies along the Sarawat escarpment northwest of Taif, while the Dam site (yellow) is located southeast of Taif in the rain-shadow zone of the Sarawat Mountains. The inset shows the position of the study area within Saudi Arabia. The non-English text appearing in the inset is part of the official institutional logo.

Al Hada:

Al Hada is situated at an altitude of 1750 m above sea level in a mountainous region 18 km northwest of Taif city, which is the most popular recreation area in southwestern Saudi Arabia (Figure 1). This site is part of the Sarawat Range, reaching elevations up to 2100 m above sea level. An escarpment road crosses the area, connecting Taif with Makkah [32]. During the study period (1990), temperatures ranged from a maximum of 36.1 °C in August to a minimum of 4 °C in January, with monthly averages between 9.2 °C and 24.3 °C. Annual rainfall was 158.1 mm, although rainfall showed high interannual variability. Fourteen years of rainfall records (1990–2003) indicate that the highest and most consistent peaks occur in April, with additional peaks frequently observed in August and November at two- to three-year intervals [31].

The site experiences frequent fog, which plays an important role in the local climate by providing supplementary moisture. This supports a high diversity of plant species, enabling *Juniperus* forests to thrive above 1500 m. Several *Vachellia* species are also present, with *Vachellia origena* being particularly abundant. During the study period, the baboon troop inhabiting the mountaintop comprised approximately 220 individuals that consistently used the same cliff areas as sleeping sites. During the daytime, the troop split into groups of varying sizes, depending on season and food availability.

Dam site:

The Dam site is a hilly and wadi-dominated area located 7 km southeast of Taif city (Figure 1). It is located in the rain shadow of the eastern Sarawat Mountains at an elevation of approximately 1500 m. During the study period in 2000, temperatures ranged from a maximum of 37.3 °C in August to a minimum of 7.3 °C in January, with monthly averages between 16.2 °C and 30.4 °C. The cumulative rainfall for that year was 136.9 mm. Over a 14-year span, annual rainfall varied widely, from 78.7 mm to 360.3 mm, with a mean of approximately 200 mm. This positioning in a rain shadow results in a much drier environment than Al Hada [31]. *Vachellia* is the dominant vegetation type at the Dam site, with *Vachellia asak* primarily occurring in the hilly areas and *Vachellia gerrardii* dominating the wadis. During the study period, the baboon troop at the Dam site consisted of approximately 475 individuals using the same sleeping site.

**Figure 2:**
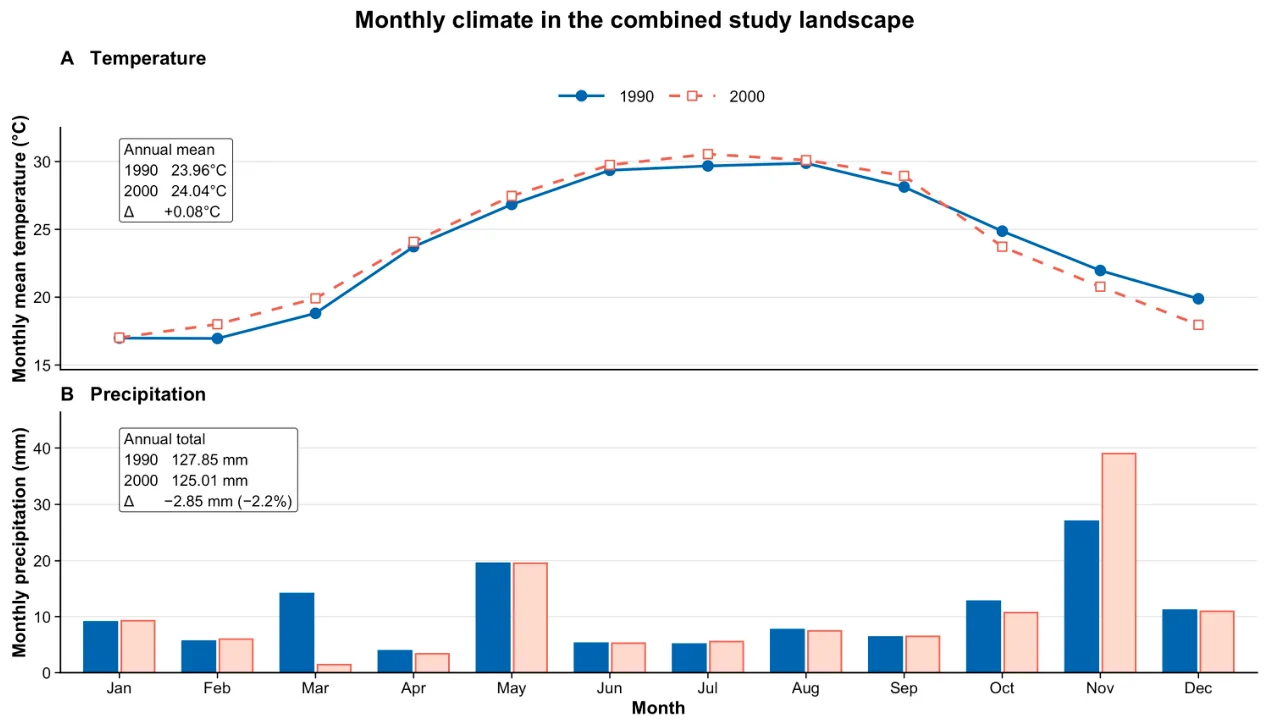
Monthly temperature and precipitation during the two study years, 1990 and 2000, across the common study landscape encompassing the Al Hada and Dam sites. The upper panel shows monthly temperature and the lower panel shows monthly precipitation; the insets summarize annual conditions.

To assess possible climatic differences associated with the ten-year separation, monthly temperature and precipitation for 1990 and 2000 were extracted at 2.5 arc-minute resolution from CRU-TS 4.09 data downscaled and bias-corrected with WorldClim 2.1 [35,36]. Both years were summarized to the same extent (21.0–21.5° N, 40.0–40.5° E), encompassing both sites. Annual conditions were broadly similar, although 2000 showed a slight increase in mean temperature from 23.96 to 24.04 °C (+0.08 °C) and a slight decrease in total precipitation from 127.85 to 125.01 mm (−2.85 mm; −2.2%), while monthly patterns showed variations (Figure 2).

### 2.2. Data Collection and Analysis

#### 2.2.1. Scan Sampling

We collected data on the hamadryas baboons at Al Hada from January to December 1990 and at the Dam site from January to December 2000. Both sites were monitored over one annual cycle using the same field methods, providing methodologically comparable datasets despite the 10-year temporal separation. The site-years are non-contemporaneous, and there is no relocation across years within either site. Our analyses were designed to describe within-year temporal variation in the proportion of visibly estrous females and black infants, not to infer stable long-term reproductive seasonality.

We determined activity profiles via scan sampling of all visible animals. Scans were taken at 15 min intervals over a period of five full days each month for one year, resulting in 720 h of observations and 40,536 records at Al Hada. Sampling was conducted during the same week each month as much as possible to maintain a relatively consistent interval between monthly sampling periods. When unfavorable weather or other logistical constraints prevented fieldwork, sampling was delayed by only a few days. At the Dam site troop, a total of 75,915 records were collected during 720 h of observation over the 12 months of 2000. The estrous status of adult females was determined by visual assessment of anogenital sexual swellings during scan samples, using established baboon field protocols. Females with fully tumescent sexual swellings were classified as estrous, as this stage generally corresponds to the periovulatory phase and heightened sexual receptivity in baboons. Black infants were recorded during scan samples based on the characteristic black natal coat coloration typical of newborn and up to six-month-old hamadryas baboons. The presence of black infants was used as an indicator of recent births.

All scans were conducted by a single observer, eliminating issues of inter-observer variability. During fieldwork, the observer recorded scan data using a voice recorder, ensuring real-time documentation under field conditions. These audio recordings were subsequently transcribed and digitized into standardized field data sheets.

#### 2.2.2. Vegetation Diversity

To quantify plant diversity and natural food availability, we conducted vegetation surveys using the line-transect method within the baboons’ home ranges. Each study site was divided into two main habitat zones—mountain slopes and valley areas—to capture ecological variation in vegetation composition. Within each zone, 5–10 transects of 100–200 m in length were established following the natural topography. Along each transect, every plant species intercepted by the line was recorded, together with its life form and abundance class. For each habitat, we calculated Shannon–Wiener diversity (H′) as

$$H^{\prime }=-\sum_{i=1}^{s}p_{i}ln\;(p_{i})$$

where $p_{i}$ is the relative abundance of species *i* based on proportional cover or occurrence frequency. These indices were used as quantitative indicators of natural food diversity in subsequent models.

#### 2.2.3. Dietary Habits

Specifically, we focused on documenting the dietary components of the baboons during the scan sampling by recording food items consumed by the individuals exhibiting feeding behavior. For each feeding record, we recorded the food item consumed, including the plant species where identifiable and the specific part of the plant being eaten (e.g., leaves, fruits, stems). This data was collected to facilitate subsequent analysis of the baboons’ dietary habits.

#### 2.2.4. Statistical Analyses of Reproductive Activity

All statistical analyses were conducted in R version 4.4.0 [37]. Because the two site-years were non-contemporaneous, and site was completely confounded with calendar year, Al Hada (1990) and the Dam site (2000) were analyzed separately. Each site-year comprised 12 monthly observations. The response variable was the monthly percentage of visible adult females classified as fully tumescent. Predictor variables included season, monthly rainfall, natural feeding records, anthropogenic feeding records, and natural food diversity. The hot season was defined a priori as May–September, corresponding to the period of highest sustained temperatures at both sites, and the cool season as October–April [38].

Two complementary modeling approaches were applied. First, Gaussian generalized linear models (GLMs) with an identity link were fitted to evaluate additive linear associations between the monthly percentage of estrous females and the predictor variables. For each site-year, the model included season and the four monthly ecological variables as additive fixed effects: Estrous-female percentage ~ season + rainfall + natural feeding records + anthropogenic feeding records + natural food diversity. Because the response was expressed as a percentage rather than as individual-level binomial outcomes, GLM coefficients were retained on the original percentage scale. The seasonal coefficient therefore represents the estimated percentage-point difference between the hot and cool periods. Model assumptions were evaluated using residual-versus-fitted and normal Q–Q plots.

Second, exploratory generalized additive models (GAMs) were fitted using the mgcv package to allow for potential nonlinear associations between estrous-female percentage and the monthly ecological predictors. GAMs used a Gaussian family with an identity link and included season as a categorical parametric term, together with separate thin-plate regression splines for rainfall, natural feeding records, anthropogenic feeding records, and natural food diversity: Estrous-female percentage ~ season + s(rainfall, k = 4) + s(natural feeding records, k = 4) + s(anthropogenic feeding records, k = 4) + s(natural food diversity, k = 4).

The basis dimension was restricted to k = 4 for each smooth to limit model flexibility given the small number of monthly observations. GAM performance was summarized using adjusted R^2^ and percentage deviance explained. Smooth terms were evaluated using their estimated degrees of freedom, approximate *F*-tests, and associated *p*-values, whereas the seasonal effect was assessed from its parametric coefficient. Because each model was based on only 12 monthly observations, all GAM-derived associations, fit statistics, and significance tests were interpreted as exploratory.

Principal component analysis (PCA) was performed using standardized ecological variables to eliminate redundant environmental variables and isolate major joint axes of variation [39]. Variables included rainfall, food diversity, anthropogenic food ratio, natural food ratio, and total food records (Table 1). PCA ordination was used to visualize ecological clustering patterns between the Al Hada and Dam troops.

## 3. Results

### 3.1. Monthly Variation in Food Availability, Rainfall, and Food Diversity

Monthly patterns of food availability and rainfall differed markedly between Al Hada and the Dam site (Table 2). Across all months, anthropogenic food availability was consistently higher than natural food availability at both sites—particularly at the Dam site. Although overall plant species diversity was higher at the Al Hada site, consistent with its greater moisture availability, the Dam site supported a higher abundance of palatable food plants for baboons.

### 3.2. Temporal Variation in the Proportion of Estrous Females

**Figure 3:**
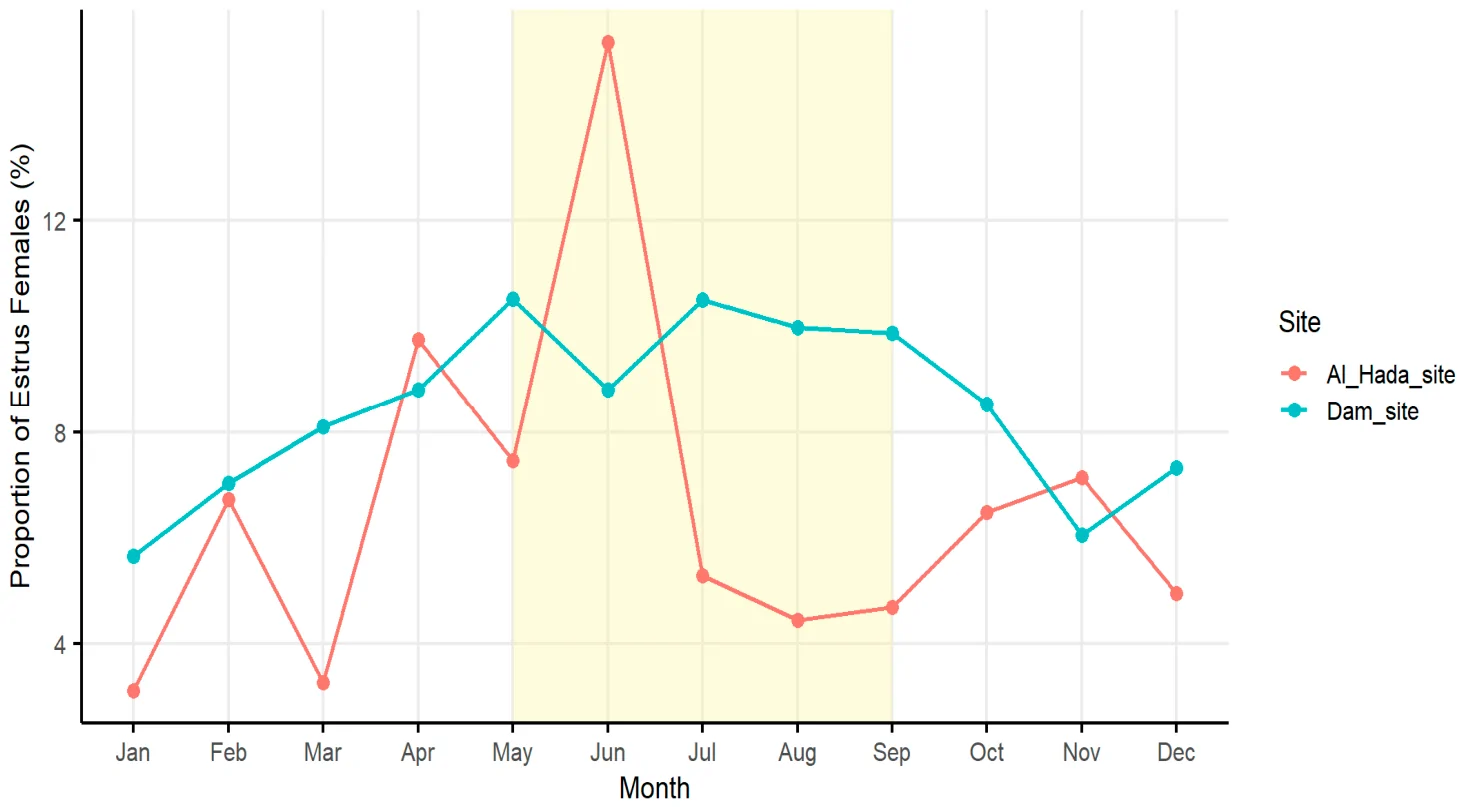
Monthly variation in the proportion of estrous females observed at the Dam and Al Hada sites. The shaded region (May–September) represents the hot season.

The proportion of estrous females showed clear within-year variation, with an observed peak during May–July at the Dam site, where values gradually increased from January and peaked during May–July (≈10–11%), followed by a decline toward November. In contrast, the Al Hada site exhibited sharper fluctuations, with a pronounced peak in June (≈15%) and lower proportions during the cooler months (Figure 3).

### 3.3. GLM and GAM Results for Predictors of Estrous Female Proportion

Dam Site

**Figure 4:**
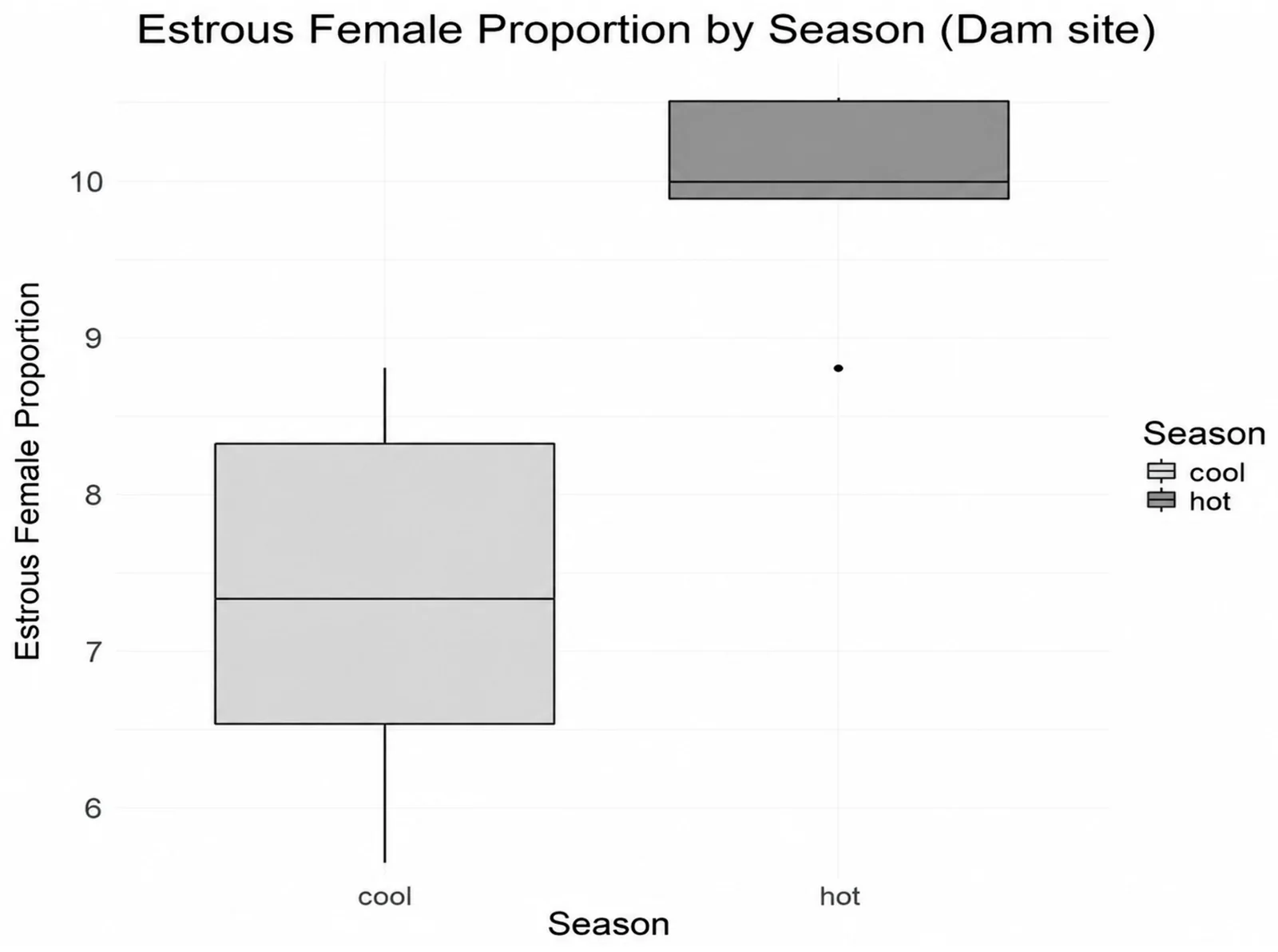
Temporal variation in the proportion of estrous females in the baboon troop at the Dam site (year 2000). Boxes indicate the interquartile range, the thick horizontal line shows the median, and whiskers extend to the minimum and maximum values within 1.5 × IQR and black dots indicate values beyond this range.

At the Dam site, the GLM identified a significant time-related effect, with higher estrous proportions during the hot months of the year (Estimate = 3.84, *p* = 0.019). Other variables were non-significant (all *p* > 0.25) (Figure 4). The GAM confirmed the importance of season (Estimate = 3.69, *p* = 0.019), explaining 80.7% of deviance (adjusted R^2^ = 0.611), with no significant smooth effects for ecological predictors (all *p* > 0.28).

We fitted a generalized additive model (GAM) using a Gaussian family with an identity link function to assess how ecological and climatic factors influence the proportion of estrous females. The model included smooth terms for natural food intake, anthropogenic food, rainfall, and natural food diversity (each with a limited basis dimension of k = 4 to prevent overfitting), along with season (hot vs. cool) as a categorical parametric term.

The intercept was estimated at 6.90 (*p* < 0.001), about three-quarters of the peak estrous value. During the hot season, the proportion increased by an average of 3.69 units (*p* = 0.019). In contrast, none of the four environmental predictors showed significant smooth effects. However, these *p*-values are approximations that do not account for the uncertainty in smoothing parameter estimation and should therefore be interpreted cautiously.

The effective degrees of freedom (edf) for all smooth terms were close to 1, indicating that the model treated these effects as approximately linear, and no strong nonlinear relationships were detected. The adjusted R^2^ was 0.611, and the model explained approximately 80.7% of the null deviance. The available GLM and GAM results for both sites are summarized in Supplementary Table S1.

In summary, this analysis demonstrates a temporal influence on estrus but finds no clear nonlinear association between the measured ecological variables and reproductive activity in the Dam site population.

Al Hada

**Figure 5:**
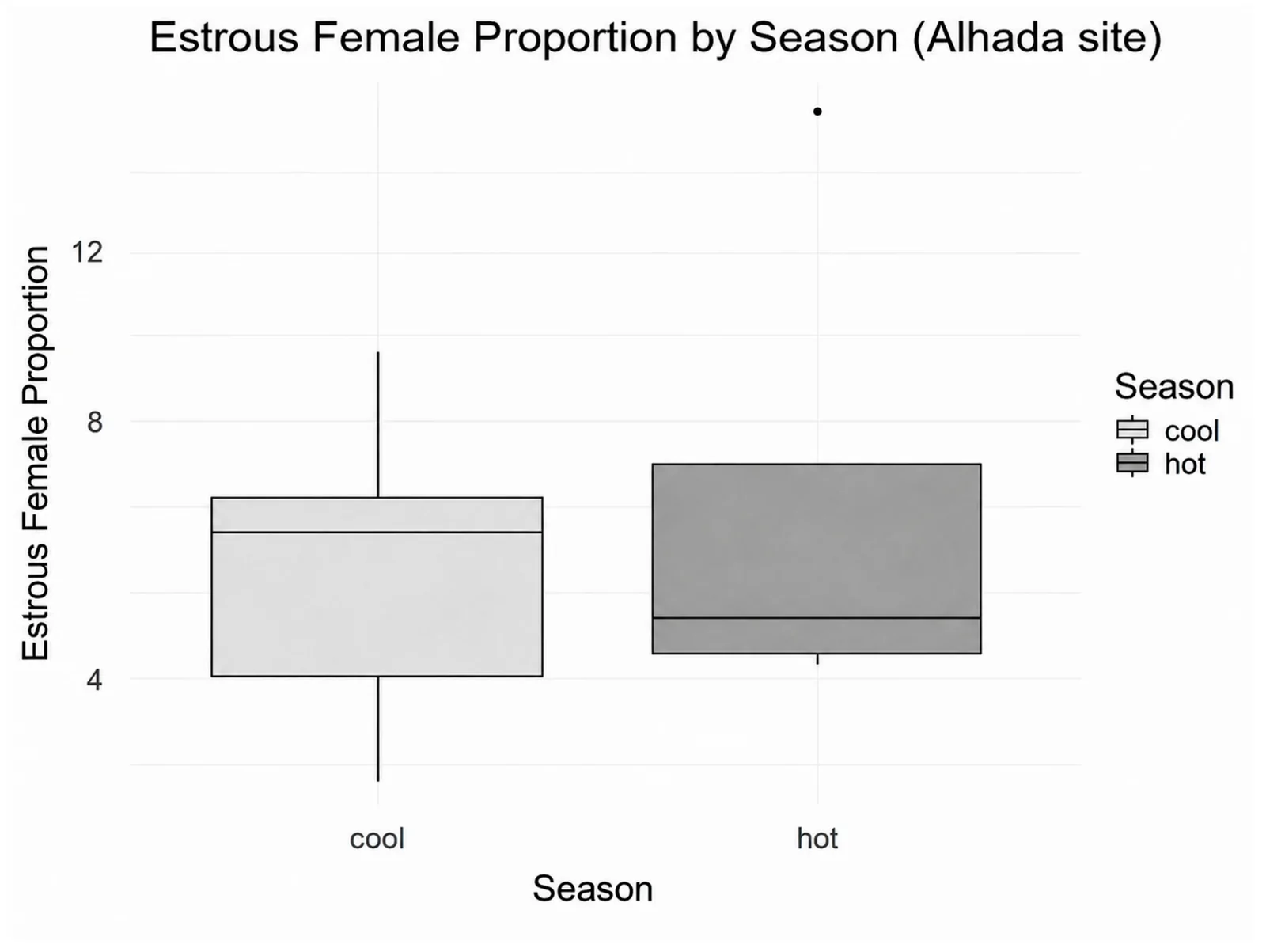
Temporal variation in the proportion of estrous females in the baboon troop at the Al Hada site (year 1990). Boxes represent the interquartile range, horizontal lines indicate medians, and whiskers extend to 1.5 × IQR Black dots indicate values beyond this range.

GLMs showed no significant linear effects of predictors on estrous proportions (season: *p* = 0.564; all other predictors: *p* > 0.5; Figure 5).

**Figure 6:**
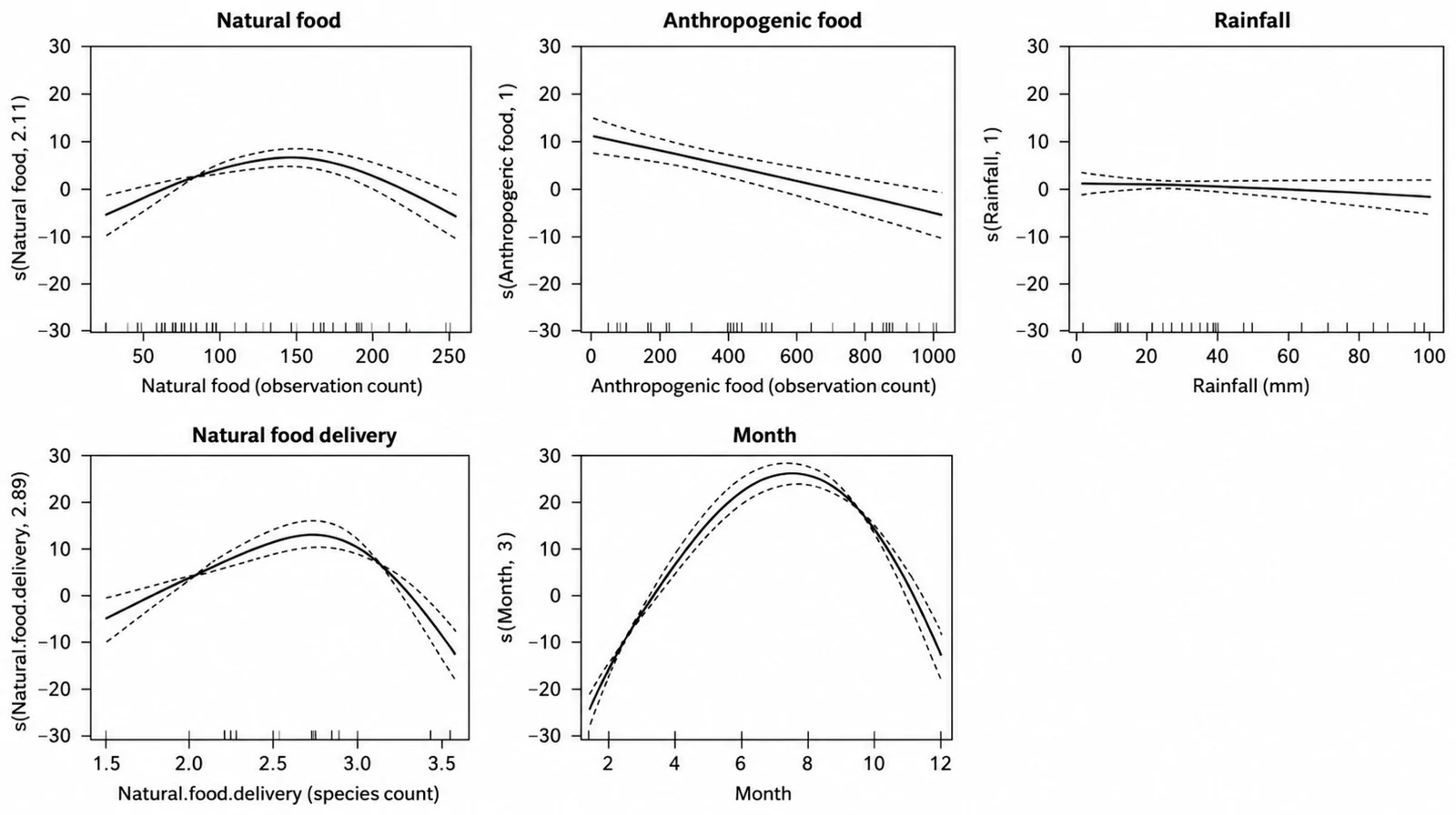
Partial effect plots from a generalized additive model (GAM) illustrating the influence of predictor variables on the proportion of estrous females in the baboon troop at the Al Hada site (1990). Each *y*-axis, s(predictor), shows the centered partial contribution of that predictor to the fitted estrous-female percentage on the model scale: values above zero indicate an above-average fitted contribution, and values below zero indicate a below-average contribution, conditional on the other model terms. Solid lines represent estimated smooth functions; dashed lines indicate 95% confidence intervals. Tick marks on the x-axes indicate observed data density.

At Al Hada, the exploratory GAM produced an apparent perfect fit (100% deviance explained; adjusted R^2^ = 1.00). Rainfall (*p* < 0.001), the proportion of anthropogenic feeding records (Ar; *p* = 0.002), and food diversity (*p* = 0.004) showed significant nonlinear associations with the proportion of estrous females. However, given the limited number of monthly observations (consistently fewer than ten, regardless of season) and the complexity of the model, these results may reflect model overfitting and should be interpreted cautiously (Figure 6). These findings suggest that within-year variation in reproductive activity at Al Hada was associated with fine-scale variation in rainfall, anthropogenic food use, and food diversity.

These results indicate that nonlinear relationships better explain variation in reproductive activity at Al Hada, with environmental variables playing a key role.

### 3.4. Temporal Variation in the Proportion of Black Infants

**Figure 7:**
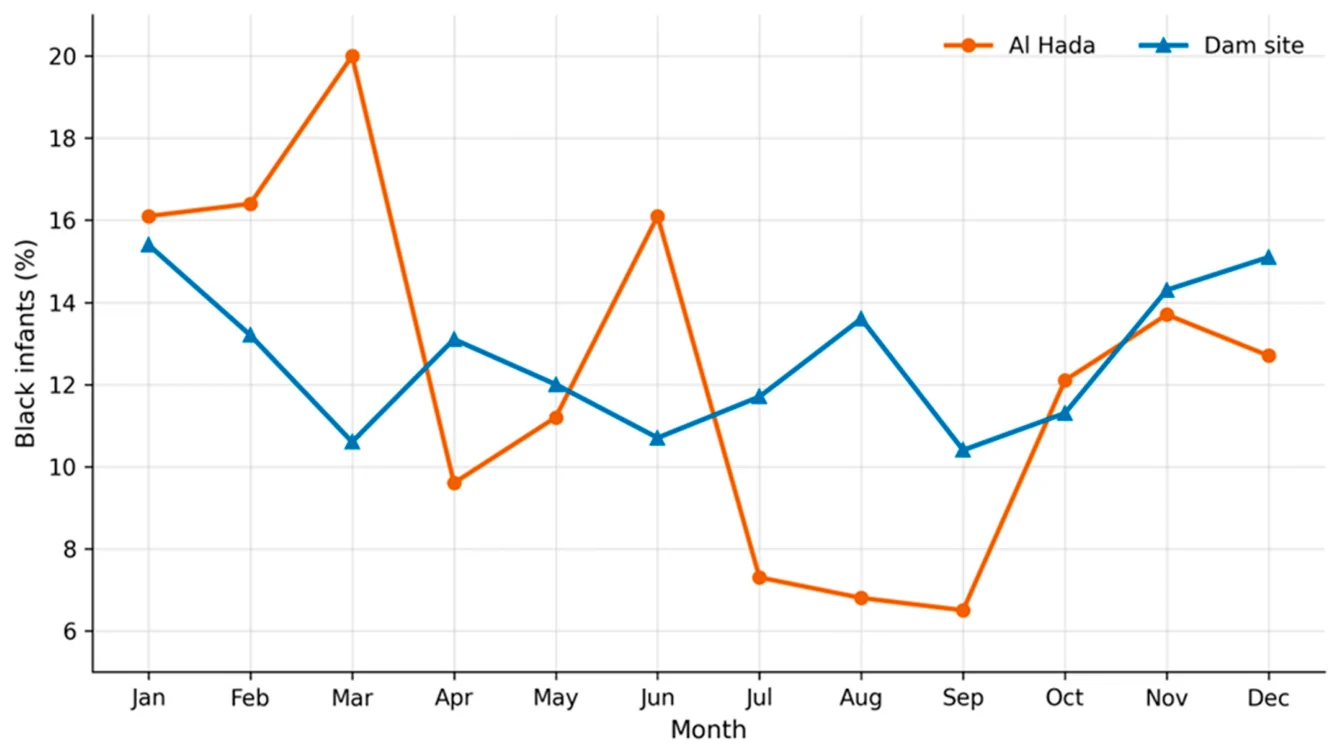
Monthly variation in the proportion of black infants in hamadryas baboon troops at the Al Hada and Dam study sites in Saudi Arabia.

The proportion of black infants varied predictably with time at each site. Greater variability was observed at Al Hada than at the Dam sight (Figure 7). At the Al Hada site, the proportion fluctuated markedly month to month, with the highest value recorded in March, followed by a sharp decline during July–September, before increasing again toward the end of the year. The Dam site showed more moderate fluctuations, and the proportion of infants peaked in the cold season and remained low during the hot season.

### 3.5. Ecological Differentiation Between Study Sites

**Figure 8:**
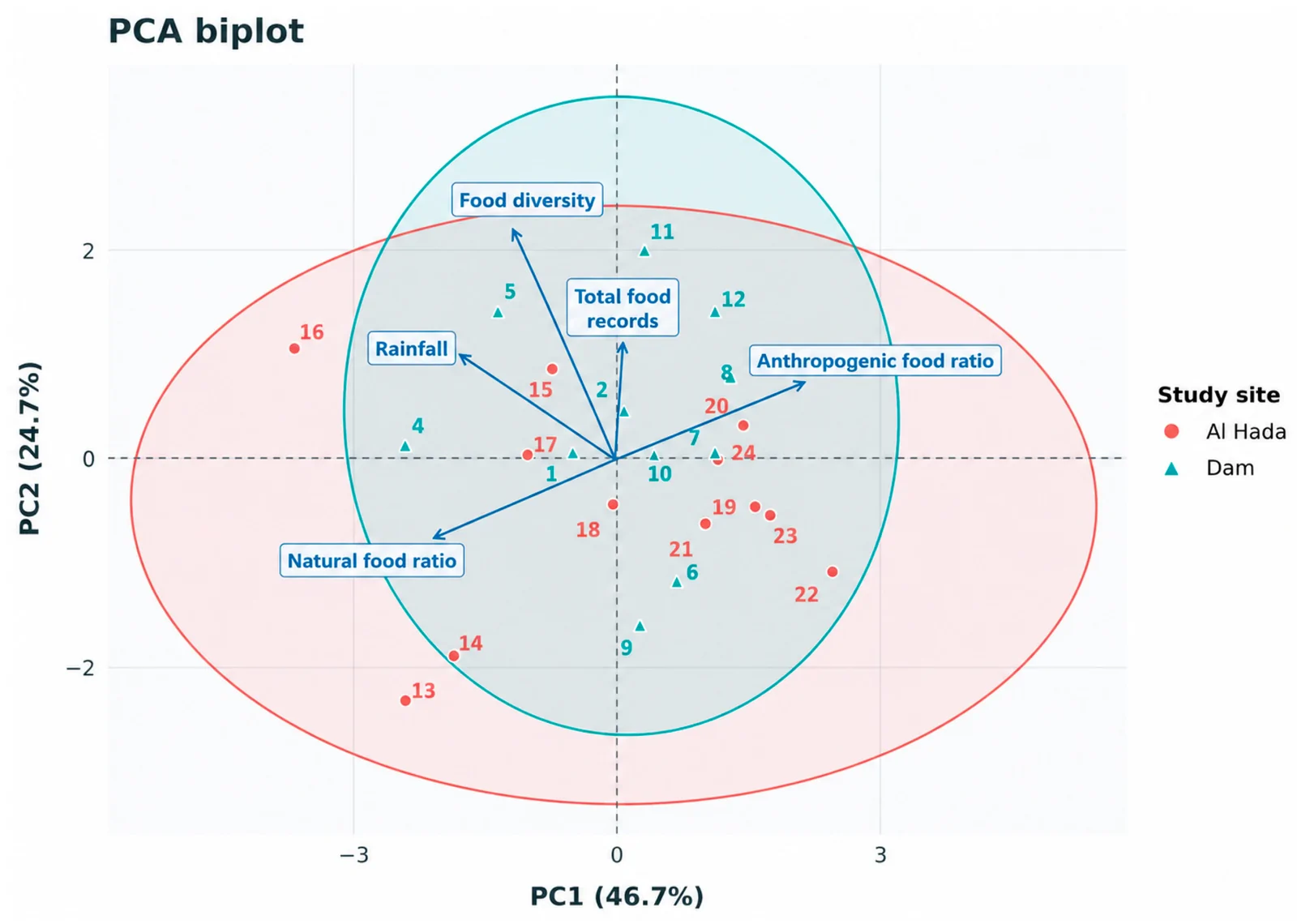
Principal component analysis (PCA) biplot showing ecological differentiation between hamadryas baboon study sites at Al Hada and Dam based on monthly environmental and feeding variables. The shaded ellipses represent the 95% confidence regions for monthly observations at each study site.

PCA revealed ecological differentiation between the Al Hada and Dam troops (Figure 8). The first principal component (PC1) explained 46.7% of the total variance, while the second principal component (PC2) explained 24.7%. Anthropogenic food intake was positively associated with PC1, whereas natural food intake, rainfall, and food diversity loaded negatively along the same axis. Samples from Al Hada were generally associated with higher anthropogenic food use, while Dam samples clustered closer to natural food consumption and rainfall-related variables. These results indicate substantial ecological differences in feeding patterns and resource use between the two troops.

## 4. Discussion

Our results show differences in the reproductive activity of female hamadryas baboons in western Saudi Arabia at two sites. At the more arid site, estrous activity and the percent of black infants were highly structured. There was a higher percentage of estrous females in the hot season and a higher percentage of black infants in cold season. At the less arid site, estrous activity, as well as the proportion of black infants, was not closely tied to the hot–cool seasonal division. Fine-scale ecological conditions, including rainfall, plant food diversity, and access to anthropogenic foods, appear to play a stronger role in shaping reproductive patterns at this site. The variability in reproduction between these sites suggests potential population-level flexibility in reproductive timing (i.e., weak taxon-specific control and fixed environmental responsiveness). This finding is important because hamadryas baboons are generally described as non-seasonal breeders. Our work reinforces evidence in support of an opposing view, arguing that the timing and the magnitude of reproductive peaks are variable and structured in the species [7,17,18]. Recent comparative work on Africa-dwelling papionins further supports this view, emphasizing that reproductive seasonality is better treated as a continuous trait rather than a simple seasonal/non-seasonal category, with substantial variation in the strength and timing of reproductive clustering among populations and species [3]. This interpretation is also consistent with studies of other cercopithecine primates showing that reproductive peaks are shaped by multiple interacting ecological and social factors rather than by a single environmental cue [5,6]. Our findings are broadly consistent with this comparative framework. For example, chacma baboons in the Okavango Delta exhibit conception peaks closely aligned with annual rainfall and food availability [10], while olive and yellow baboons show varying degrees of seasonality depending on habitat productivity and climatic predictability [8,42]. Together, these comparisons suggest that reproductive timing in baboons is shaped by local ecological conditions rather than by a fixed species-wide pattern, and our study extends this perspective to hamadryas baboons in the Arabian Peninsula.

A key limitation of this study is the ten-year gap between sampling at Al Hada (1990) and the Dam site (2000). The ten-year separation between the two study periods introduces the possibility that interannual climatic variation contributed to the observed differences between the troops. However, comparison across a fixed regional extent showed no marked difference in the annual climatic values: 2000 was only 0.08 °C warmer and received 2.85 mm less precipitation, equivalent to a 2.2% reduction, compared with 1990. Thus, it is unlikely that the study gap could have influenced plant phenology, food availability, and reproductive timings. Another limitation of this study is that the survey reflected a single annual cycle at each site. Many species exhibit variability in reproduction across years and multiyear data reflects that best.

Variation in the proportion and timing of estrous females reflects the strong biological coupling between baboon reproduction and energetic conditions. Rainfall, food diversity, and anthropogenic subsidies influence female reproductive readiness and mating synchrony. Dezeure et al. (2024) found that, across papionins, greater year-to-year rainfall unpredictability was associated with lower or absent reproductive peaks, suggesting that flexible reproductive phenology may be favored where seasonal resource peaks are unreliable [3]. This framework is particularly relevant to our study because the two sites differ not only in rainfall patterns and vegetation, but also in the predictability and accessibility of anthropogenic foods. Previous studies have shown that access to human food subsidies can affect the onset of estrus and reduce interbirth intervals by shortening infant rearing times in urban baboon populations, thereby accelerating reproductive turnover [33,43,44]. Such plasticity in breeding phenology likely enhances reproductive output under variable environments but may also intensify human–baboon conflict when reproductive peaks coincide with periods of increased reliance on anthropogenic resources [33].

The temporal signal observed at the Dam site could be linked to the rain-shadow conditions, characterized by low and unpredictable rainfall, sparse vegetation, and a narrow window of primary productivity. Although vegetation cover is limited, drought-tolerant *Vachellia* species and ephemeral herbs provide resources following spring rains, creating a brief period during which females can accumulate energy reserves. While rainfall was not a significant linear predictor in our models, its effects were partly captured by the hot season, when vegetation quality is highest, a pattern commonly observed in arid and semi-arid systems [10,45].

Similar dynamics have been documented in other primates and large mammals inhabiting seasonal environments, where conception is timed so that energetically demanding stages of pregnancy and lactation coincide with periods of improved forage availability [2,11]. In hamadryas baboons, Polo and Colmenares (2016) showed that even in a food-provisioned colony, ovarian activity retained moderate seasonality, suggesting that intrinsic physiological rhythms—potentially linked to photoperiod or temperature—interact with nutritional constraints to shape reproductive timing [19]. The Dam site population may thus rely on such intrinsic cues to synchronize reproduction during the narrow ecological window that maximizes offspring survival.

The Al Hada population exhibited no discernible temporal pattern. Reproductive activity followed short-term ecological fluctuations. This highland site, influenced by frequent fog and higher rainfall, supports greater plant biomass and diversity, providing a relatively stable baseline of natural food availability. Our GAM results indicate that estrous activity increased following rainfall events and periods of elevated plant food diversity, consistent with an opportunistic response to transient improvements in nutritional conditions.

This pattern aligns with long-standing evidence that food availability is a key proximate driver of primate reproduction [8,46]. Short-term increases in energy intake can rapidly restore ovulatory function and increase the probability of conception, particularly in species with flexible life histories [2,45]. At Al Hada, rainfall-triggered vegetation flushes corresponding to reproductive pulses could reflect a different tracking mechanism. Similar breeding patterns have been reported in other primates inhabiting heterogeneous or unpredictable environments [12,47]. Predation risk could influence ranging, refuge use, vigilance, and feeding time, with indirect effects on energy balance and reproductive behavior [48]. However, predator abundance and anti-predator responses were not systematically measured in this study; therefore, this explanation remains tentative.

Anthropogenic food availability influenced reproductive patterns, particularly at Al Hada, where tourist feeding and access to human-derived foods are reliable. Our findings indicate that months with higher anthropogenic food use were associated with increased estrous proportions, suggesting that calorie-rich subsidies can buffer females against natural food shortages. Similar effects have been documented in semi-provisioned baboon populations, where access to human foods alters ranging behavior, energy budgets, and reproductive output [43].

At the Dam site, however, provisioning was more intermittent than at Al Hada, and synchronization with food provisioning was not apparent in the time series. Consequently, baboons at the Dam site may rely more strongly on seasonal cues than on anthropogenic buffers. Importantly, even at Al Hada, baboons did not become fully dependent on human foods; when rainfall increased natural food availability, reliance on anthropogenic resources declined [31]. This pattern underscores the continued importance of natural vegetation in regulating reproductive ecology, even in human-modified landscapes.

Patterns in the proportion of black infants broadly complemented the estrous data and provided additional insight into the temporal distribution of recent births across the two populations. At the Dam site, the relatively moderate yet persistent fluctuations in black infant proportions suggest that births occurred in higher proportions in the cooler months. In contrast, the Al Hada population showed stronger short-term variation, including pronounced peaks and declines across months, indicating more localized clustering of births. This temporal pattern is consistent with the approximately six-month transition from black natal coat to brown fur, because infants observed in March would progressively leave the black-infant category by August to September. Therefore, the late-summer decline cannot be interpreted directly as a reduction in births. These differences likely reflect the contrasting ecological dynamics between the two habitats. However, changes in the proportion of black infants require cautious interpretation. An increase is consistent with recent births, whereas a decline may result from infants maturing and losing their black natal coat, infant mortality, or temporary non-detection when infants are concealed by their mothers or subgroups are out of view. We therefore treat the proportion of black infants as an index of the presence of young infants rather than a direct estimate of birth or survival rates [3,7,18]. The contrasting patterns between sites may reflect differences in birth timing, but this interpretation requires confirmation through individual-based, multiyear monitoring of births, coat transitions, survival, and detectability.

Together, these findings highlight the reproductive plasticity of hamadryas baboons. Flexibility could confer a strong fitness advantage by aligning energetically costly reproductive stages with periods of resource abundance [45]. Indeed, Polo and Colmenares (2016) demonstrated higher failure rates for conceptions that were poorly timed relative to environmental conditions, suggesting strong selection for reproductive synchrony with favorable ecological windows [19].

From a management perspective, these results have direct implications for mitigating human–baboon conflict. Periods of elevated births substantially increase energetic demand and risk-prone foraging behavior. These phases are often associated with intensified use of anthropogenic food sources, increased roadside activity, and crop raiding [7,33,34,43]. By identifying ecological and temporal predictors of reproductive peaks, managers can anticipate periods of heightened conflict risk and implement proactive measures such as waste control, targeted deterrence, or public awareness campaigns before conflict escalates.

## 5. Conclusions

This study offers a management-relevant insight into the peak reproductive activity of hamadryas baboons in response to ecological variability and human-derived food subsidies in western Saudi Arabia. To the best of our knowledge, this is the first study to show that hamadryas baboon reproduction is linked to the time of year in harsher, arid landscapes or opportunistically regulated by rainfall, food diversity, and anthropogenic resources in more productive habitats. Our findings move beyond describing within-year reproductive peaks and identify the factors influencing reproductive timing. The interactions between short- and longer-term environmental fluctuations in shaping this pattern are an area needing further development. Given the escalating human–baboon conflict in Saudi Arabia, the findings of this study directly influence wildlife management decisions concerning the timing and types of interventions [30]. Periods of elevated estrous signal forthcoming peaks in births, energetic demand, and risk-prone foraging behavior, all of which are known to exacerbate human–baboon conflict incidents [33,34]. Managers can therefore use these results to anticipate high-risk windows and proactively time interventions before conflict intensifies.

More broadly, this study documents reproductive plasticity in hamadryas baboons and suggests that anthropogenic food influences population processes that remain a control challenge for wildlife authorities. Future research could extend this framework through multiyear monitoring, integration of hormonal and body-condition measures to quantify energetic thresholds for conception, and linkage of reproductive timing with movement and conflict data to improve early-warning systems for human–baboon interactions. Such integrative studies are essential for developing evidence-based coexistence strategies that balance primate conservation with human safety in rapidly changing landscapes.

## Figures and Tables

**Table 1:** Predictor variables included in GLMs and GAMs, with descriptions and data sources.

Variable	Description	Source	Unit/Measurement
Natural food intake	Frequency of foraging on wild plants recorded during scan sampling	This study (behavioral observations, 15-min scan intervals)	Number of records of natural food intake during the observation period
Anthropogenic food	Frequency of feeding on human-provided food (e.g., garbage, crops, handouts)	This study (behavioral observations, 15-min scan intervals)	Number of feeding records on anthropogenic food per observation period
Rainfall	Monthly total precipitation at each study site	National Center for Meteorology	mm per month
Natural food diversity (Shannon H′)	Vegetation diversity index calculated from line-transect surveys in baboon home ranges	This study (field vegetation surveys, 5–10 transects/site)	Dimensionless index (H′)
Season	Temporal factor representing climatic seasonality and phenological cycles	Derived from monthly climate	[38]

Visualization of predictor effects on estrous proportions was conducted with ggplot2, generating scatter plots and fitted trend lines. Model performance was evaluated using adjusted R^2^ values and deviance explained. The significance of individual predictors in GAMs was assessed using approximate significance tests for smooth terms *(F*-tests). To explore temporal effects further, we conducted post hoc pairwise comparisons using Tukey’s honestly significant difference (HSD) tests [40,41] under both two-season and four-season frameworks, adjusting for multiple comparisons. Results are presented with estimated effect sizes, standard errors, and *p*-values.

**Table 2:** Monthly variation in ecological and anthropogenic variables recorded from systematic scan sampling of hamadryas baboon troops at Al Hada and the Dam site. For each month, values represent the total number of scan records in which baboons were observed feeding on natural food items and anthropogenic food sources, along with corresponding monthly rainfall (mm) and natural food diversity indices for each site.

Month	Natural Food (No. of Records)		Anthropogenic Food (No. of Records)		Rainfall (mm)		Natural Food Diversity Indices	
	Al Hada	Dam Site	Al Hada	Dam Site	Al Hada	Dam Site	Al Hada	Dam Site
1	216	541	205	1345	8.7	0	2.3	3.01
2	283	580	338	1900	0	0	2.54	3.16
3	98	302	395	1315	29.7	19.7	3.44	3.44
4	161	1003	221	1156	75.2	11.6	3.61	3.45
5	185	385	514	1146	36.4	54.7	2.73	3.14
6	86	407	315	1344	0	0	2.86	1.75
7	66	245	743	1617	0	12.5	2.25	2.14
8	55	219	842	2372	0	1.3	2.82	2.75
9	42	624	314	1436	7.9	0	2.28	1.51
10	16	327	639	1290	0	3.4	1.5	2.84
11	71	250	882	2029	0	37.7	2.07	3.14
12	79	197	718	2064	0	2	2.72	3.28

## Data Availability

The original contributions presented in this study are included in the article. Further inquiries can be directed to the corresponding authors.

## Supplementary Materials

The following supporting information can be downloaded at: https://www.mdpi.com/article/10.3390/ani16172724/s1. Table S1: Summary of the generalized linear model (GLM) and generalized additive model (GAM) results for predictors of the proportion of estrous females at Dam Site and Al Hada Site.
