# Supplementary material for: Ecological Drivers of Breeding Peak in Hamadryas Baboons (Papio hamadryas) Across Two Contrasting Habitats in Saudi Arabia
Source: Animals (Basel). 2026 Sep 2;16(17):2724. doi: 10.3390/ani16172724 (PMC13564629; doi:10.3390/ani16172724)
Supplement: Supplementary file 1 [file animals-16-02724-s001.zip › animals-4514920-supplementary.pdf]

animals

MDPI

Supplementary Table S1

Table S1. Summary of the generalized linear model (GLM) and generalized additive model (GAM) results for predictors of the proportion of estrous females at Dam Site and Al Hada Site.

Site	Model	Predictor/term	Estimate	edf	F	p-value	Model fit
Dam Site	GLM	Season: hot vs. cool	3.84	NR	NR	0.019	NR
Dam Site	GLM	Other ecological predictors	NR	NR	NR	All $p > 0.25$	NR
Dam Site	GAM	Season: hot vs. cool	3.69	Parametric term	NR	0.019	Adjusted $R^2 = 0.611$; deviance explained = 80.7%
Dam Site	GAM	Natural food intake	NR	≈1	NR	>0.28	Adjusted $R^2 = 0.611$; deviance explained = 80.7%
Dam Site	GAM	Anthropogenic food	NR	≈1	NR	>0.28	Adjusted $R^2 = 0.611$; deviance explained = 80.7%
Dam Site	GAM	Rainfall	NR	≈1	NR	>0.28	Adjusted $R^2 = 0.611$; deviance explained = 80.7%
Dam Site	GAM	Natural food diversity	NR	≈1	NR	>0.28	Adjusted $R^2 = 0.611$; deviance explained = 80.7%
Al Hada Site	GLM	Season: hot vs. cool	NR	NR	NR	0.564	NR
Al Hada Site	GLM	Other ecological predictors	NR	NR	NR	All $p > 0.5$	NR
Al Hada Site	GAM	Rainfall	NR	NR	NR	<0.001	Adjusted $R^2 = 1.00$; deviance explained = 100%
Al Hada Site	GAM	Anthropogenic food	NR	NR	NR	0.002	Adjusted $R^2 = 1.00$; deviance explained = 100%
Al Hada Site	GAM	Natural food diversity	NR	NR	NR	0.004	Adjusted $R^2 = 1.00$; deviance explained = 100%
